# A Beginner’s Guide to Cardiac Optical Mapping

**DOI:** 10.1007/s13239-026-00863-9

**Published:** 2026-10-15

**Authors:** Katy A. Trampel, Binjie Li, Sharon A. George, Igor R. Efimov

**Affiliations:** 1 Department of Biomedical Engineering, Northwestern University, Chicago, IL USA; 2 Department of Pharmacology and Chemical Biology, the University of Pittsburgh, 200 Lothrop Street, Pittsburgh, PA 15261 USA; 3 Department of Medicine, Northwestern University, 303 E Superior Street, Chicago, IL 60611 USA

**Keywords:** Optical mapping, Cardiac electrophysiology, Arrhythmia, Action potential, Calcium transient, Experimental design

## Abstract

Optical mapping is a high spatiotemporal resolution imaging technique used to study cardiac electrophysiology by recording fluorescent signals that correspond to changes in transmembrane potential, intracellular calcium, and metabolic activity. This review provides a comprehensive guide for beginners, covering the fundamentals of optical mapping system components, including dye selection, optical filters and mirrors, camera specifications, and system configurations. A range of tissue preparations, from single cells to whole hearts, is described, with attention to how preparation type affects experimental design, perfusion methods, and system setup. Pharmacological and computational strategies to reduce motion artifacts are discussed. Electrical stimulation protocols, such as pacing and restitution mapping, are described in the context of arrhythmia induction and rate dependent electrophysiological properties. Technical considerations including system maintenance and troubleshooting are also addressed. Finally, the review outlines current limitations, including dye cytotoxicity, tissue viability, and photobleaching, and explores future directions such as genetically encoded indicators and oxygen-enriched perfusates. This guide aims to equip new users with the foundational knowledge needed to design, perform, and interpret optical mapping experiments.

## Cardiac Electrophysiology Techniques

Each heartbeat originates as an electrical impulse in the sinoatrial (SA) node. These electrical impulses then travel through the myocardium triggering the opening and closing of numerous ion channels, allowing ions such as K^+^, Cl^-^, Na^+^, and Ca^2+^ to move across the membrane, causing a sequence of rapid changes in electrical potentials across cardiomyocyte membranes, which is defined as an action potential [1, 2]. The initiation of an action potential, in turn, releases Ca^2+^ ions from the extracellular space and sarcoplasmic reticulum into the cytoplasm, which binds to cytoskeletal structures and produces contraction [3]. The effective functioning of ion channels and pumps involved in electrical excitation and contraction requires energy in the form of ATP generated by cardiac metabolism. Thus, metabolism, electrical excitation, and contraction in the heart are interdependent processes, and well-tuned metabolism-excitation-contraction coupling (MECC) is critical for proper cardiac function [4]. Cardiac diseases and drugs can severely impact various aspects of cardiac function, and these perturbations can be life-threatening. Cardiac electrophysiology is routinely investigated in drug toxicity studies and disease models [5, 6]. Techniques currently employed to assess cardiac electrophysiology include patch clamping, electrode arrays, and cardiac imaging methods such as optical mapping.

The patch clamp technique has been widely employed to effectively monitor almost any type of cell's single or collective ion channel activities [7]. Conventional patch clamp approaches, using negative pressure to seal a micropipette onto a single cell membrane, can record responses of individual or ensemble channels by inside-out, outside-out, or whole-cell configurations [8, 9]. Subsequent advances, such as automated and planar-array-based patch clamp recording, significantly improved throughput and efficiency [10–12]. However, some limitations, such as invasiveness, still exist [13].

The use of electrodes is a common approach to study electrophysiology. Traditional electrical mapping places electrodes at various locations of the heart to record electrical activities and infer conduction patterns [14]. Clinical electroanatomical mapping is an advanced form of it, by incorporating the 3D structure of the heart, which better assists clinicians in identifying sites for ablation or surgery. Microelectrode array (MEA) is another common system to acquire extracellular or intracellular electrical signals from multiple cells at relatively high spatiotemporal resolution [15, 16]. Currents through ion channels generate an electrical field that can be detected and measured by electrodes in an MEA system. Recent advances in bioresorbable and flexible MEAs present possibilities of applying MEAs to monitor electrophysiology of dynamic hearts spatiotemporally in situ [17–19], though the current resolution is still limited due to the size of microelectrodes and interconnects.

Optical mapping is an imaging method that is currently used to measure a variety of cardiac physiological processes, including transmembrane potential, calcium transients, and metabolic markers – nicotinamide adenine dinucleotide (NADH) and flavin adenine dinucleotide (FAD) levels. Optical mapping can be used to image cell, tissue, and organ-level models, which makes it a highly versatile tool. Recently, we have developed a contact optical mapping which can be used in vitro and in vivo, including open chest or implantable device configurations [20]. This review will focus on the basics of an optical mapping protocol, system considerations, and troubleshooting.

## Designing an Optical Mapping System

Optical mapping is a highly versatile technique that can be applied to record multiple physiological parameters from many different types of tissue preparations. Thus, the experimental design will vary depending on the research question. Below, we will go over some considerations for designing and setting up an optical mapping study.

### Dyes and Reporter Genes

One of the first considerations when preparing for an optical mapping experiment is to determine the parameter that will be measured. The most commonly measured parameters are transmembrane potential, intracellular calcium, electromechanics, mitochondrial membrane potential, NADH, and FAD. While some parameters, such as NADH and FAD, can be measured without external fluorescent probes [21–23], others require dyes or genetically encoded reporters.

The right choice of dye will depend on the parameter(s) being measured and the properties of the dye. For cardiac electrophysiology measurements, dyes with fast kinetics, high signal-to-noise ratio (SNR), and significant fractional fluorescence change (ΔF/F, change in fluorescence intensity in response to change in parameter being measured) are preferred [24, 25]. Many commonly used voltage-sensitive dyes in optical mapping, such as Di-4-ANEPPS, RH237, and Di-4-ANBDQBS, operate based on electrochromatic (also known as potentiometric) mechanisms. These dyes bind to the extracellular region of the plasma membrane and contain chromophores with large ground-to-excited state dipole changes [26–28]. Changes in transmembrane potential alter the charge distribution of the dye, resulting in subtle, voltage-dependent shifts in their excitation and/or emission spectra. These spectral shifts are linearly proportional to changes in transmembrane potential. Importantly, the magnitude and polarity of the signal depend on the specific excitation and emission wavelengths selected. Therefore, filter choice is critical: selecting wavelengths on the steepest part of the dye's spectral response curve enhances the voltage sensitivity of the optical system. The precise alignment of the detection system with the dye’s voltage-sensitive spectral region is essential for achieving optimal signal fidelity and sensitivity.

Dyes could also be single-wavelength non-ratiometric dyes or dual-wavelength ratiometric dyes. In ratiometric imaging, fluorescence signals are normalized using a ratio of two measurements, which helps compensate for photobleaching, uneven dye loading, and motion artifacts [29]. There are two types of ratiometric measurements: excitation ratiometry [30, 31] and emission ratiometry [29]. In excitation ratiometry, the dye is excited by two different wavelengths of light, and the emitted signals are collected separately [30, 31]. This approach is useful when the emission spectrum is narrow or when it is important to separate excitation-dependent effects. In cardiac optical mapping, this is particularly important for dyes that undergo electrochromic shifts, such as voltage-sensitive dyes, because their spectral response to transmembrane voltage changes occurs primarily in the excitation band [27]. By capturing signals at different excitation wavelengths while keeping emission constant, excitation ratiometry enhances voltage sensitivity, improves quantification accuracy, and reduces artifacts from tissue autofluorescence, overlapping emissions, and motion. In contrast, emission ratiometry involves excitation by a single wavelength of light, and emission at two different wavelengths is collected separately [29]. This method is often preferred in cardiac optical mapping when high-speed imaging is required, as it permits continuous excitation and simultaneous dual-channel detection with minimal temporal delay. This is particularly advantageous for capturing rapid events such as action potential upstrokes or intracellular calcium transients, where precise temporal and spatial resolution is critical. Because both emission signals are acquired concurrently, emission ratiometry also minimizes signal distortion from motion artifacts, ensuring greater fidelity in the mapping of fast electrical and calcium dynamics in beating cardiac tissue. The most commonly used fluorescent probes for optical mapping of various cardiac parameters, including their excitation and emission wavelengths, advantages, disadvantages, loading methods, and practical considerations, are summarized in Table 1. It is important to note that dyes should be sonicated before use, especially if directly injected into the heart, to break up fine particles and improve staining.

**Table 1.** Fluorescent probes used in cardiac optical mapping [32, 33, 110–118]

Parameter	Dye	Excitation/ Emission Peaks (nm)	Advantages	Disadvantages	Loading Characteristics/Practical Considerations
Transmembrane potential	Di-4-ANEPPS	470/620	• Rapid fluorescent response (femtoseconds scale) • High ΔF/F = 7–10%	• Blue-green excitation limits imaging depth • High autofluorescence from NADH/ hemoglobin • Rapid internalization; phototoxicity and bleaching	• Rapid uptake (5–10 min) • Compatible with for slices, EHTs, small animals • Short imaging window due to internalization • Requires flow slowdown or recirculation • Cytotoxic with prolonged exposure
	Di-8-ANEPPS	456/618	• Higher membrane retention vs. Di-4-ANEPPS • Similar voltage sensitivity to Di-4-ANEPPS	• Similar depth and autofluorescence limitations as Di-4-ANEPPS • Slightly slower temporal response than Di-4-ANEPPS	• More lipophilic; slower internalization compared to Di-4-ANEPPS • Single-pass loading possible, recirculation preferred
	Di-2-ANEPEQ	477.5/624	• Good signal-to-noise ratio • Fast response (sub-ms) • Excellent dye for microinjection • Significant tissue penetration was applied externally	• Lower ΔF/F sensitivity (≈2–10% per 100 mV) • Blue-green excitation limits imaging depth • Limited published cardiac data • Washes out quickly	• Requires recirculation • Similar loading to other ANEP dyes
	Di-4-ANBDQBS	531/722	• Red-shifted excitation for deeper imaging • High ΔF/F = 10–18% • Long signal retention (80–210 min) • Compatible with blebbistatin	• Slightly slower response than Di-4-ANEPPS • More expensive than Di-4-ANEPPS	• Strong membrane binding allows long-duration imaging • Single-bolus effective • Low cytotoxicity, no re-dosing required
	Di-4-ANBDQPQ	541/733	• Similar to ANBDQBS in performance and spectrum • Excellent retention and red-shifted spectra	• Less commercially available	• Single bolus effective • Low toxicity • No flow modification needed
	RH237	520/770	• Historically popular for V_m_ mapping • High fluorescence yield (ΔF/F = 6%)	• Green excitation limits imaging depth • Rapid photobleaching and internalization • High autofluorescence	• Often requires re-dosing due to washout after a couple of hours • Cytotoxic with prolonged exposure • Flow must be slowed to ensure uptake
	Fluovolt	520/551	• High voltage sensitivity (~27% ΔF/F per 100 mV) • Sub-ms response time	• Rapid washout in 3D/perfused tissue • Limited to short-duration experiments • Moderate toxicity in cell culture	• Applied via static incubation (15–30 min) • PowerLoad kits enhance loading efficiency • Best suited for cultured cells and thin tissue slices
Intracellular Calcium	Rhod2-AM	553/576	• Red emission (good for multiplexing) • Fast kinetics	• Partial mitochondrial localization • Limited tissue penetration • Moderate toxicity • Non-ratiometric	• Single administration • Mixed with Pluronic F-127 to increase cell loading • Superfusion or perfusion (5–30 min) • Allow 5–10 min washout of unbound dye
	Fluo-4	494/516	• Bright signal • Fast kinetics • Rapid loading	• Blue excitation limits depth • Non-ratiometric • Prone to photobleaching/washout	• Mixed with Pluronic F-127 to increase cell loading • Use static incubation, superfusion, or perfusion • Compatible with monolayers, slices, whole hearts
	Indo-1	388/400, 475	• Ratiometric: corrects for motion, loading, photobleaching • Fast response	• Requires UV excitation (cytotoxic, limited depth) • Dual-emission optics required	• Mixed with Pluronic F-127 to increase cell loading • Incubate or perfuse for 45–60 min • Suitable for monolayers, slices, small hearts
	Fura-2	340,380/510	• Ratiometric: corrects for motion, loading, photobleaching	• Requires UV excitation (cytotoxic, limited depth) • Dual-excitation optics required • Risk of organelle compartmentalization	• Mixed with Pluronic F-127 to increase cell loading • 45–60 min incubation needed • Works best in thin slices or isolated cells
Mitochondrial Membrane potential	tetramethylrhodamine methylester (TMRM)	559/589	• Reversible, non-quenching at ≤ 200 nM • Fast and sensitive to ΔΨm changes • Red-shifted = good for multiplexing	• Cytotoxic at high concentrations • Leaks if mitochondria depolarize	• Continuous perfusion or brief bolus • Wash-out if ΔΨm changes • Load time 15 – 30 minutes • Compatible with cell monolayers, tissue slices, and whole heart preparations

More recently, genetically encoded fluorescent reporters have been developed and used in optical mapping [32, 33]. These genetically encoded voltage or calcium indicators (GEVIs and GECIs, respectively) are expressed in transgenic mice or are virally transfected in tissue of other species. Recent advances in this field have produced GEVIs and GECIs with sensitivity and kinetics that are on par with the organic dyes typically used in optical mapping. These reporters can also alleviate the limitations of using fluorescent dyes, such as bleaching, dye washout, and toxicity. In addition to these genetically encoded reporters, optical mapping also leverages the intrinsic autofluorescence molecules, such as NADH and FAD [4, 34]. These endogenous fluorophores require no staining and provide valuable real-time insights into mitochondrial function and tissue metabolism, critical to excitation-contraction coupling. Table 2 summarizes the excitation and emission characteristics of commonly used GEVIs, GECIs, and intrinsic autofluorescent molecules relevant to cardiac optical mapping. This is a rapidly evolving field that produces novel genetically encoded indicators for various important biological parameters.

**Table 2:** Genetically encoded fluorescent reporters and intrinsic autofluorescence sources used in cardiac optical mapping [22, 119]

Type	Reporter	Excitation/Emission Peaks (nm)
Genetically Encoded Voltage Indicator (GEVI)	VSFP2.3	440/480
	ArcLight	490/520–535
	FlicR1	560/630
Genetically Encoded Calcium Indicator (GECI)	GCaMP8	470/510
	R-CaMP2	565/583
	R-GECO2L	563/581
	NIR-GECO	678/704
Combined GEVI and GECI	CaViar	488,635/510,710
Intrinsic Autofluorescence	NADH	330–360/440–470
	FAD	360–465/520–530

A final note on choice of fluorescent dyes and reporters: when using combinations of dyes and/or genetic reporters, it is essential to confirm that the emission wavelengths of the different agents do not overlap. For example, one commonly used combination for dual optical mapping of transmembrane potential and intracellular calcium is RH237 and Rhod2-AM, respectively. These two dyes have overlapping excitation wavelengths, but their emission wavelengths do not significantly overlap. This facilitates using a single excitation light source to excite both dyes and allows separating the emitted light from the two dyes into two different light paths while recording the optical signals. Overlapping emission wavelengths among dyes used together can cause crosstalk between the recorded signals and affect the collected data and its interpretation. Several other dye combinations are also suitable for dual-parameter optical mapping, depending on the spectral characteristics of the optical system and experimental goals. These include Rhod 2 and di-4-ANBDQPQ [35], Indo 1 and di-4-ANEPPS [36, 37], and Fura 4F and di-8-ANEPPS [26].

### Optics

**Fig. 1:**
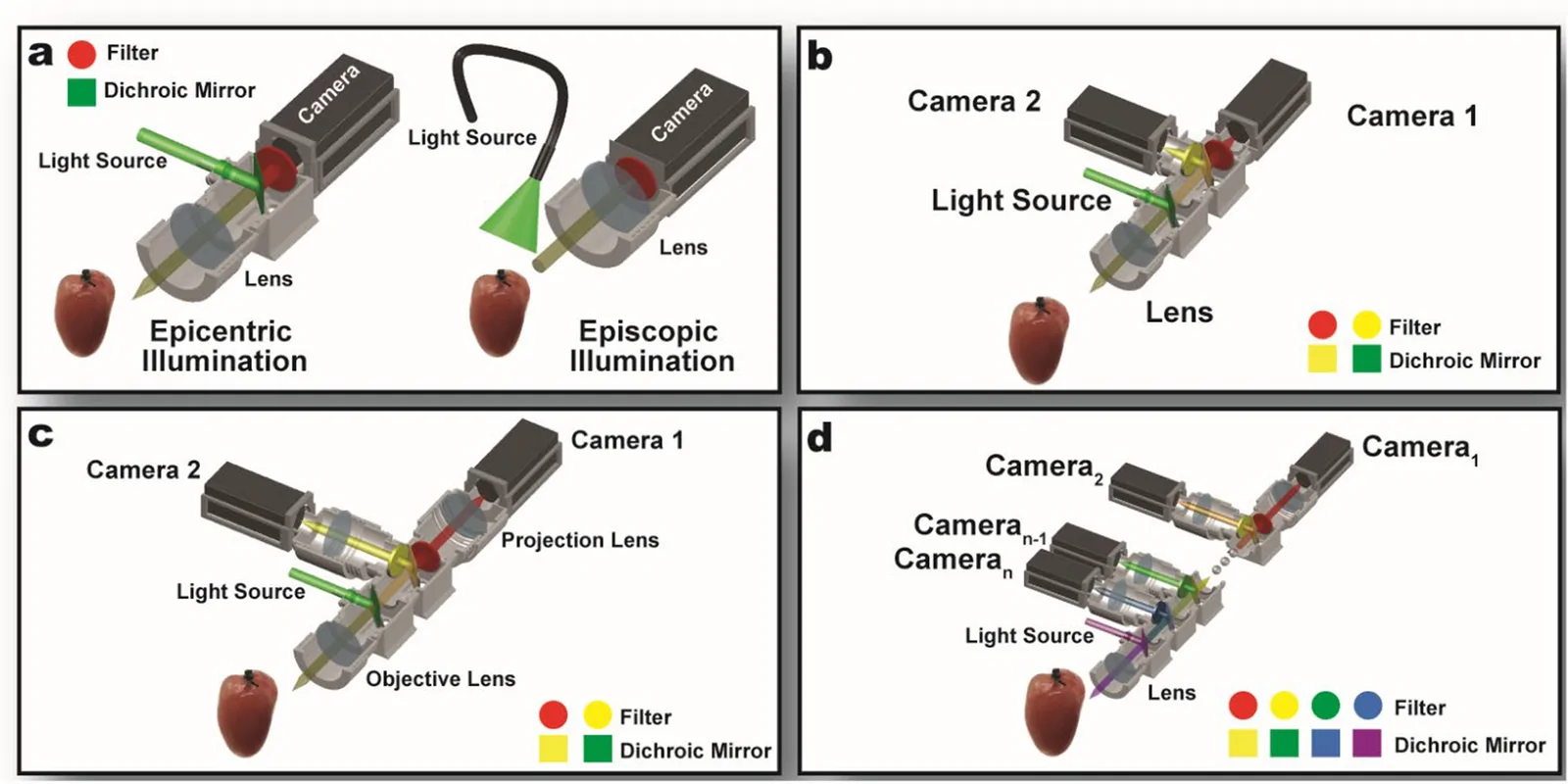
Optical mapping system configurations. [38] **A** Illustrations of optical mapping systems with epicentric and episcopic excitation light illuminations are shown. (**B**-**C**) Single-lens and tandem-lens two-camera systems to measure two physiological parameters are illustrated. **D** An illustration of the potential to record multiple parameters of cardiac physiology in tandem lens configuration is illustrated Adapted from Cathey et al 2019.

Optical components required for the system include excitation light sources and light guides, optical filters, dichroic mirrors, and lenses. Halogen or high-power LED light sources are typically used for the excitation of the fluorescent probes in optical mapping studies. The use of collimator lenses and excitation filters to focus the light at the correct wavelength onto the tissue preparations is recommended. Light sources can be used in epicentric or episcopic illumination modes as illustrated in Figure 1a [38]. In the epicentric approach, light is guided into a filter cube that is part of the optical mapping setup, reflects off a dichroic mirror, passes through the objective lens, and precisely illuminates the field of view. In the episcopic approach, the excitation light guides are attached to the sides of the optical mapping setup and are manually adjusted so that they illuminate the field of view.

When choosing the filters and dichroic mirrors for the optical mapping system, the excitation and emission spectra of the fluorescent probes being used should be considered, and the selection should prevent the overlap of signals from different dyes in any given camera. Lastly, the lenses used in the optical mapping system are also crucial in determining the signal quality and spatial resolution. In our experience, plan apochromatic lenses with high numerical aperture and short working distances work well for this application, because they maximize light collection. The magnification required of the lens will depend on the size of the field of view to be imaged. Additionally, the format of the lens should be carefully chosen to match the size of the photodetector sensor; mismatches may lead to vignetting (if the lens format is too small) or wasted light collection (if the lens format is too large), both of which degrade optical performance and sensitivity.

Lenses can be used in two configurations: single and tandem lenses. In single lens configuration (Figure 1b), the optical mapping system consists of a single objective lens connected to filter(s) and camera(s). In the tandem lens configuration (Figure 1c ,d), each light path has two infinity-corrected lenses facing each other, an objective and a projection lens. The objective lens (closer to the tissue) collects the light emitted by the dyes in the tissue and projects it at infinity. This infinity-corrected light is then passed through filters and dichroic mirrors as needed to separate and guide the light to different cameras. The projection lens collects this filtered light and focuses it onto the camera sensor. The use of a tandem lens configuration has been reported to improve SNR [39]. The infinity-corrected light path between the objective and projection lenses also allows multiple cameras to be placed along the light path, allowing for multi-parametric imaging [4, 38].

### Cameras

An advantage of optical mapping over electrode array mapping of cardiac electrophysiology is the high spatial resolution offered by the former technique. Furthermore, in order to faithfully record the fast changes in cardiac electrophysiology during each heartbeat, it is important to have high temporal resolution. Therefore, the cameras used in these systems should have high spatiotemporal resolution [39]. In order to achieve high spatial resolution, temporal resolution is typically sacrificed and vice versa. We recommend a minimum of 500 frames per second (fps) temporal resolution for electrophysiological studies.

### System Configurations

Once all the above components of the optical mapping system have been acquired, the system can be assembled in one of the following configurations, depending on the application. Optical mapping systems can be set up to map multiple parameters, multiple fields of view, or for panoramic imaging.

For multi-parametric optical mapping, single or multi-camera systems can be used. In a single camera, multi-parametric optical mapping system, each alternating frame records signals of a different parameter [40]. The excitation light wavelength must be different for each parameter and must switch between the different frames such that it matches the temporal resolution of the camera. In a multi-camera, multi-parametric optical mapping system, one camera is used for each parameter being recorded (Figure 1c, d). In this case, a single or multiple excitation light source can be used to excite the various fluorescent probes, and the total emitted light is collected and separated into different light paths, and each light path ends in a camera. This approach is most routinely used in dual and, more recently, triple parametric optical mapping [4, 41]. If multiple cameras are used, then all cameras must be spatially and temporally aligned such that each camera captures signals from the same field of view at the same instant of time. Multi-camera system processors and software typically have the capability to perform this spatio-temporal calibration before the start of the experiment. Systems that can simultaneously control up to four cameras are currently available [4, 42].

For multiple fields of view imaging, different cameras and optics units face each tissue surface that needs to be imaged. For example, the anterior and posterior surfaces of a heart or the epicardial and endocardial surfaces of a wedge preparation can be imaged simultaneously using this setup [43]. In this system configuration, even though each camera and optics unit is physically separate from each other, it is critical that the cameras be temporally synchronized to ensure time-aligned signals from the different surfaces of the same tissue. These cameras do not necessarily need to be controlled by the same processor. In some setups, using separate processors can even improve total recording bandwidth.

Panoramic imaging is an extension of the multiple fields of view imaging. In this instance, multiple cameras positioned at fixed overlapping angles, or a single camera orbiting the heart, are used to acquire signals from various views of the heart, enabling reconstruction of electrical activity across the epicardial surface. A geometric model of the heart’s surface is typically acquired, and the mapping data from different viewpoints are merged using texture mapping to create a unified dataset. Panoramic imaging has only been reported for single-parameter optical mapping to date [42, 44–47]. Finally, there are several other optical mapping system configurations that are less frequently used, such as alternating transillumination [48, 49] and optrodes for transmural imaging [50–52].

## Designing an Optical Mapping Study

An optical mapping study needs to consider several other factors besides the optical mapping system. These include the type of tissue to be used, how this tissue will be kept viable during the experiment, means to reduce noise and artifacts in the optical signals, and auxiliary physiological parameters that should be measured. This section of the review will discuss these factors.

### Tissue Preparations – Cells to Organs

Optical mapping has been performed on cells, tissue, and whole hearts from various species. Optical mapping allows for the high-resolution imaging of cellular physiology on a microscopic level. Single-cell optical mapping was first performed in isolated guinea pig ventricular cardiomyocytes to investigate cellular excitation [53]. Monolayers of cultured human and neonatal rat cardiomyocytes have also been optically mapped to record transmembrane potential [54, 55].

At the tissue level, optical mapping has been performed on engineered heart tissues/muscles (EHT/M), organotypic cardiac slices, and isolated, superfused, or perfused preparations from different regions in the heart. EHTs are usually fabricated in a hydrogel with stem cell-derived cardiomyocytes or primary cells such as neonatal rat cardiomyocytes [56–58]. For EHTs, optical mapping has been applied to investigate tissue maturity or their ability to emulate adult myocardium and to study human cardiac diseases *in vitro* [59]. Organotypic cardiac slices are thin (maximum of 400 mm thickness) slices of cardiac tissue with their extracellular matrix (ECM) and electrical and mechanical junctions intact. Due to the thinness of the organotypic cardiac slices, numerous slices can be collected from the same heart, and this platform has emerged as a high-throughput preclinical drug and disease testing model [60, 61]. Unlike cardiomyocyte isolation, organotypic slices are not subject to enzymatic damage [62]. However, mechanical damage can occur during the slicing process. Slicing tangentially to the epicardium has been recommended to reduce mechanical damage during the slicing process [63]. Isolated tissue preparations can be prepared from different regions of interest in the heart, such as the atria, ventricles, nodal tissue, and right ventricular outflow tract. While some isolated tissue preparations, such as mouse right and left atria with or without the sinoatrial and atrioventricular nodes, can be prepared by simple superfusion, owing to the thin nature of these regions [64, 65], others require coronary perfusion. Coronary perfused intact tissue preparations, such as ventricles, septum, right ventricular outflow tract, and even atria from larger hearts, are also routinely used in electrophysiological assessments [43, 66].

Whole-heart optical mapping provides an intact organ-level assessment of electrophysiology from the epicardial surface using one or multiple cameras. A two-camera configuration can capture the anterior and posterior of the heart [47]. A four-camera configuration captures a more complete spatial arrangement. Panoramic imaging captures multiple points-of-view of the heart via multiple cameras and mirrors, which is imperative for larger hearts (i.e., rabbit, pig, human) [47, 67]. While panoramic imaging allows for spatiotemporal rich information, it is limited by the cost of the system and data analysis complexities. More recently, optical mapping of beating *in situ* swine hearts has become possible, allowing the assessment of electrical activity in a physiologically perfused environment with preserved autonomic and hemodynamic influences [68].

### Perfusion

During an optical mapping experiment, the heart must be supplied with nutrients and oxygen, and waste products must be removed to keep the heart viable. To meet these requirements, whole blood and buffered perfusates are standardly circulated through the heart. In the case of whole blood perfusion, a donor animal supplies blood to an experimental heart that is being optically mapped. In a donor animal system, the donor animal is anesthetized, and the large arteries and veins are cannulated [69–71]. The donor blood is circulated from the donor animal to the experimental heart and then returns to the donor. Whole blood can also be perfused through the reservoir. Autologous blood from the experimental animal, often mixed with a crystalloid solution, can also be used. However, there may be challenges with oxygenation and subsequent frothing, although these can be mitigated by using membrane oxygenators rather than gas bubbling systems [72]. It is also important to note that hemoglobin in blood absorbs light at wavelengths commonly used for fluorescence imaging, which can reduce the signal-to-noise ratio and must be considered when designing experiments involving blood perfusion.

Buffered fluid perfusates emulate the ionic concentrations found in blood. Common buffered perfusate compositions and concentrations are described in Table 3. Krebs-Henseleit (KH) solution has a high calcium chloride concentration because its original formula does not consider the amount of calcium chloride in the blood bound to proteins [72]. Tyrode’s solution has a slightly different ion composition and is favored for optical and electrophysiological studies due to ionic concentrations. Many laboratories customize perfusate formulations depending on species, tissue type, and experimental goals. For example, the addition of substrates like insulin or fatty acids may be appropriate for metabolic studies. Glucose is typically used as the primary energy source in buffered perfusates, as it is water-soluble, unlike fatty acids, which are hydrophobic and prone to foam formation [72]. If fatty acids are used to better mimic physiological conditions, antifoaming agents or membrane oxygenators are added to the perfusion system. Insulin is sometimes incorporated in perfusates due to diabetic levels of glucose but is rarely used in studies due to the complex effects of insulin [72]. Bicarbonate buffered solutions should be oxygenated with carbogen (95% oxygen / 5% carbon dioxide) to maintain stable physiological pH. Perfusates should be maintained at physiological pH (7.4) and temperature (37⁰C).

**Table 3:** Common buffered perfusates and their compositions

Perfusate	NaCl mM)	NaHCO₃ (mM)	KCl (mM)	MgSO₄ (mM)	KH₂PO₄ (mM)	C₆H₁₂O₆ (mM)	NaH₂PO₄ (mM)	CaCl₂ (mM)	MgCl_2_ _(mM)_	Albumin (%)	C_3_H_3_NaO_3 (mM)_	Insulin (U/L)
Krebs-Henseleit Solution	118.50	25.00	4.70	1.20	1.20	11.00	NA	2.50	NA	NA	NA	NA
Modified Krebs-Henseleit solution	118.00	24.00	3.30	1.20	1.20	10.00	NA	2.00	NA	2.00	2.00	NA
Crystalloid perfusate	115.00	26.00	3.30	1.80	1.20	11.00	NA	1.25	NA	NA	NA	10.00
Tyrode’s Solution	130.00	25.00	4.75	1.20	NA	5.00	NA	1.25	NA	NA	NA	NA
Modified Tyrode’s Solution	130.00	24.00	4.00	NA	NA	5.60	1.20	1.80	1.00	NA	NA	NA

Several practical considerations improve success and reproducibility in heart perfusion experiments. Anticoagulants such as heparin are typically administered to the animal (or added to blood or buffer) prior to heart excision to prevent thrombus formation. Immediately following excision, the heart may be flushed with cold cardioplegic solution to arrest contraction and minimize ischemic damage. This is particularly useful when tissue is sectioned into organotypic slices or dissected into wedges. After cannulation and start of perfusion, the heart should be allowed to equilibrate for at least 10–15 minutes. The exact time depends on species, temperature, cardiac region, and use of electromechanical uncouplers. In some cases, such as with left ventricular organotypic tissue slices and wedges, exogenous electrical pacing must be applied throughout the optical mapping experiment. Additionally, in some cases, if electrical activity does not spontaneously respond, such as in whole heart preparations, external stimulation, such as pacing or defibrillation, may be needed to reinitiate beating.

It is essential to prevent the introduction of air into the coronary circulation, as this can cause immediate ischemia. Bubble traps filled ~ ¾ with perfusate should be placed in the tubing line to intercept air, and the tubing downstream should be carefully inspected before beginning perfusion. If the perfusate is recirculated, a filtration system must be included to prevent tissue debris or precipitates from reentering the heart and causing occlusions. These measures are critical to maintaining tissue viability and ensuring high-quality optical signals throughout the experiment.

#### Retrograde Perfusion

Retrograde perfusion, also called Langendorff perfusion, is a technique that perfuses the heart via coronary flow through the cannula inserted in the aorta [71]. The aortic valve is closed due to the pressure from retrograde perfusion down the aorta. Perfusion flows into the coronary arterial vasculature through the coronary ostia. Then it circulates through the coronary bed and the coronary veins to supply the heart with the perfusate. A typical Langendorff perfusion system consists of a cannula that adequately fits the aorta but must be carefully positioned to avoid extending too far into the ascending aorta, as this may compromise the aortic valve or occlude the coronary ostia, impeding perfusion. If the aortic valve is insufficient or leaks under perfusion pressure, retrograde flow into the left ventricle may occur. In such cases, a vent or pressure relief cannula inserted into the left ventricle via the apex or left atrium may be used to prevent left ventricular distension and damage. Additional components of a Langendorff system include bubble traps to prevent air embolism, heat exchangers and/or hot plates to keep the perfusate at 37ºC, and a tissue bath that could be used to house the tissue being optically mapped. Perfusates are circulated once through the heart, and the effluent is either discarded or can be recirculated after reoxygenation. Langendorff perfusion can be accomplished either in the constant pressure or constant flow modes [71]. Constant pressure perfusion is achieved by positioning the perfusate reservoir at a known distance above the heart, also known as gravity perfusion or by manually/automatically adjusting the flow rate on a peristaltic pump to maintain physiological pressure (60 – 100 mmHg). Constant flow uses a peristaltic pump to provide fixed circulation, such as superfusion for EHT, atrial preparations, and organotypic slice mapping. While constant flow allows for a consistent flow rate, the heart cannot autonomously adjust the flow rate under different physiological conditions.

#### Anterograde Perfusion

Anterograde perfusion, or working heart perfusion, replicates *in vivo* blood flow [71]. A cannula is inserted into the aorta for retrograde perfusion until the heart is stabilized. Then, a second cannula is placed in the left atrium through the pulmonary vein. The perfusate is pumped into the left ventricle and ejected into the aortic cannula, where coronary perfusion is returned to an oxygenating reservoir for reperfusion. While working heart perfusion is more complex, it is advantageous to study mechanical activity, metabolism, and oxygen consumption.

### Electromechanical Uncoupling

The heart is an electrically and mechanically active organ that pumps blood throughout the body. Excitation-contraction-coupling (ECC) describes the process of electrical activation (action potential) triggering force generation (contraction). While optical mapping aims to capture the electrical activity of the heart, the mechanical contraction of the heart produces motion artifacts in the resting and repolarization phases of the optical action potential. Therefore, it is necessary to induce excitation-contraction uncoupling to accurately quantify electrophysiologic parameters. This was first achieved by mechanical restriction of the beating heart through epicardial contact with the optical window of the tissue bath that houses the heart [73]. Care must be taken to ensure that the pressure applied to push the heart against the optical window does not cause a local ischemic region due to reduced coronary flow.

Pharmacological methods are now commonly used for excitation-contraction uncoupling. Commonly used electromechanical uncouplers include myosin II inhibitors 2,3-butanedione monoxime (BDM)/diacetyl monoxime (DAM) and blebbistatin, as well as cytochalasin D, which impairs actin filament polymerization. While BDM effectively performs mechanical uncoupling of the heart, it has been shown to reduce action potential duration in rabbit, swine and sheep [74, 75]. Similarly, cytochalasin D has been reported to induce action potential duration prolongation, alter action potential morphology, and membrane hyperpolarization in mouse hearts [74, 75]. On the other hand, blebbistatin has proven to be a better alternative to BDM and cytochalasin D because it does not have such severe effects on cardiac electrophysiology [76]. Blebbistatin, an adenosine triphosphate (ATPases) inhibitor associated with a class of myosin II isoforms in the actin-detached state, was first used at concentrations of 5 − 10 μM and, more recently, has been demonstrated to be non-toxic at concentrations up to 25 μM [76–79]. However, blebbistatin takes longer to achieve effective uncoupling compared to BDM, typically requiring 15–45 minutes of perfusion before complete suppression of contraction is observed. This time is dependent on the species and experimental preparation. Special handling precautions are necessary for blebbistatin, as it is light-sensitive and inactivated by high-energy excitation light (i.e. ultraviolet or blue) [76, 80]. Solutions should be protected from ambient light, and imaging systems using ultraviolet or blue light excitation should be avoided or minimized. Some studies in rabbit hearts report significant electrophysiological effects of blebbistatin, including action potential duration modulation [81]. Despite these reports, blebbistatin has become the most popular electromechanical uncoupler in optical mapping studies [82]. However, a limitation of blebbistatin is its phototoxicity, intrinsic fluorescence, and high cost. Recently, light-insensitive derivatives of blebbistatin have been synthesized to overcome some of these limitations. Para-nitro blebbistatin is less cytotoxic, phototoxic, and fluorescent than blebbistatin but has poor water solubility [82, 83]. Para-aminoblebbistatin is less cytotoxic, phototoxic, and fluorescent, and has improved water solubility compared to blebbistatin and para-nitro blebbistatin but is more expensive [84]. Both para-nitroblebbistatin and para-aminoblebbistatin have not been tested for optical mapping.

Motion tracking algorithms to reduce motion artifacts in optical signals are of interest since they do not modulate cardiac electrophysiology, as is the case with pharmacological electromechanical uncouplers. The two primary categories of motion tracking are optical flow and feature tracking techniques [85]. Optical flow algorithms track every pixel in a recorded video to track displacement of tissue, whereas feature-tracking algorithms track individual points of interest. While these approaches have great benefits, they too have limitations. For example, high frame rates used during optical mapping lead to large data files that can be complex to process and analyze. Additionally, motion tracking assumes consistent fluorescent intensity over time, and this assumption is breached due to unequal dye distribution and dye washout over the course of an optical mapping experiment. Another approach to reduce motion artifacts is the excitation or emission ratiometry techniques, described above. Using ratiometric approaches, motion artifacts, inhomogeneous dye distribution, and the effects of inhomogeneous illumination are attenuated when the ratio of the two signals is calculated [86]. Motion tracking and ratiometry techniques have been combined to further minimize motion artifacts using fiducial markers or marker-free motion tracking [87, 88].

### Electrical Stimulation

Depending on the tissue preparation being used in the study, there may or may not be an intrinsic rhythm or the spontaneous beating of the heart tissue. In stem cell-derived engineered heart tissues, automaticity has often been observed owing to the immaturity of cardiomyocytes [89]. Similarly, in the case of whole hearts or other preparations where the nodal tissue (sinoatrial or atrioventricular nodes) is intact, the preparation may exhibit intrinsic rhythm. In a whole heart, this electrical signal starts at the sinoatrial node, propagates through the atria, through the atrioventricular node, His Bundle, and Purkinje fibers to the ventricle. However, the rate of the intrinsic rhythm varies between different tissue preparations and also over time in the same *ex vivo* preparation [90]. Since there are several rate dependent electrophysiological parameters that are measured by optical mapping, it is important to control the rate at which the heart beats between different test conditions. This is done by applying external electrical stimuli to pace the tissues at varying rates.

#### Pacing

Electrical stimulation by applying current or voltage, is performed using a pacing electrode typically made of platinum or silver. Pacing can be applied by placing the pacing electrode in contact with the tissue (point stimulation) or by placing the electrode in the tissue bath near the tissue (field stimulation). In point stimulation, the electrode is typically made of fine insulated wires where the insulation is striped only at the ends in order to prevent electrical shorting. The pacing electrode can be unipolar (one electrode on the surface of the heart and a reference electrode in the tissue bath) or bipolar electrode (two electrodes on the surface of the heart). In field stimulation, larger electrodes, typically in the shape of plates are positioned near the tissue, on either side, and submerged in the bath perfusate. The application of electrical field between these plates will stimulate the tissue.

The rate, pulse width, and pulse amplitude of the pacing stimuli can be varied. At the start of every experiment, the pulse amplitude at which the electrical stimulation is captured 1:1 by the tissue is determined. This is called the threshold of capture. The tissue is then stimulated at 1.5x or 2x the threshold of capture for the rest of that experiment. The rate is determined by the species being studied. For example, mouse hearts which have faster heart rates are paced faster than a human heart. The pulse rate is also varied in experiments to study the frequency-dependent properties of the tissue. Some electrophysiological parameters, such as action potential duration (APD) and conduction velocity (CV) adapt to pacing frequency. This property, which is dependent on the duration of the diastolic interval preceding a given heartbeat, is called electrical restitution [4, 91, 92].

#### Steady-state Restitution

In steady-state restitution protocols, a train of electrical pulses at a fixed rate (S1) is first applied [93]. The rate of the S1 pulses should be chosen such that 1:1 capture is still observed and no beat-beat variations in measured parameters are present. A fixed number of S1 pulses is followed by a single S2 pulse. The parameter (APD, CV, etc.) is measured from the beat that originates from this S2 pulse. The S1S2 coupling interval is then shortened in steps of 1-5 ms and the protocol is repeated until the S2 pulse is not captured. The data is plotted with the measured parameter on the y-axis and the preceding diastolic interval (sometimes basic cycle length is also used here) on the x-axis. When performing optical mapping with steady-state restitution protocol, ensure that the last few S1 pulses and the S2 pulse are captured in the optical recording to be able to measure electrical restitution.

#### Dynamic Restitution

In dynamic restitution protocol, a train of S1 pulses is delivered to the tissue at a given rate, and the electrophysiological parameter is determined for the last several beats triggered by these S1 pulses [94]. Optical recordings of these last few beats should be acquired to determine electrical restitution. This protocol is then repeated while increasing the rate of the S1 pulse train (shorter basic cycle length or duration between pulses) until loss of 1:1 capture of the electrical stimuli.

#### Arrhythmia Inducibility

Arrhythmia inducibility in cardiac tissue can be determined by applying burst pacing, S1S2, S1S2S3 or S1S2S3S4 pacing [95]. In burst pacing, a short electrical pulse train of S1 pulses is applied at a very fast rate, close to the effective refractory period of the tissue. The rate of the burst train can be increased in repetitions of this protocol. In the S1S2, S1S2S3 andS1S2S3S4 pacing, a train of S1 pulses is applied at a rate where 1:1 capture and no beat-to-beat variations in electrophysiological parameters are observed. This is followed by one to three premature pulses, an S2, an S3, and an S4 pulse. The coupling between each of these pulses is varied and shortened in repetitions of the protocol until loss of capture or arrhythmias are triggered. In the absence of an arrhythmogenic substrate, the heart typically returns to intrinsic rhythm at the end of each repetition of these protocols. In hearts with arrhythmogenic substrates, an arrhythmia such as tachycardia or fibrillation is triggered.

### Acquisition of Additional Physiological Signals

In addition to recording the pacing stimuli described above, it could be beneficial to monitor temperature, pressure, and electrocardiograms (ECG) during an optical mapping experiment to determine the physiological state of the tissue during the study. Therefore, a data acquisition unit capable of recording at least four channels of analog data is required: 1) electrical pacing channel, 2) ECG channel, 3) temperature channel, and 4) pressure channel. The ECG is recorded by placing electrodes in the bath to record volume-conducted or pseudo-ECGs. The electrical pacing channel and pseudo-ECG channel allow the user to determine if the heart is being paced at the set rate during an experiment. Temperature is essential to monitor during an optical mapping experiment because when temperature is too low or too high, it can modulate ion channel functions and electrophysiology and can lead to arrhythmias. Low temperature (less than 35⁰C) is associated with bradycardia [72], while increased temperature (greater than 39⁰C) can cause tissue damage [72]. In whole heart preparations, coronary perfusion pressure should be maintained at physiological pressure (60 – 100 mmHg). Low pressure (less than 60 mmHg) results in poor perfusion to the heart that can result in ischemia [72]. Elevated pressure (greater than 100 mmHg) causes vasoconstriction that reduces coronary flow [96, 97].

## Optical Mapping Data Analysis

Several groups have developed custom analysis software to analyze optical mapping data. Of these, Rhythm software is published under an open-source license and is available to all users at www.github.com/optiocardiography [4]. While this section focuses on defining physiological parameters derived from optical mapping signals, we refer readers interested in detailed signal preprocessing, including filtering, normalization, and drift correction, to the comprehensive review by Laughner et al., which thoroughly addresses these topics [98]. Below, we describe the various parameters that can be measured from the optical signals of various physiological functions measured by optical mapping.

### Transmembrane Potential

The change in transmembrane potential over a single heartbeat result in an action potential. The action potential consists of a sharp upstroke or depolarization, which is followed by a repolarization phase, the morphology of which varies depending on the species. From the depolarization phase, the duration of the upstroke or rise time (RT), and conduction velocity can be measured. Rise time is typically determined as the interval between 10 to 90% of the upstroke amplitude to exclude artifacts due to noisy signals. Conduction velocity can be determined by several methods. Bayly’s method is a commonly used approach [99]. In this method, activation times are determined as the time of the maximum first derivative of the upstroke of the action potential. Activation times are plotted along the x- and y-axis of a surface with known interpixel resolution, and a parabolic surface is fitted to it. The largest directional derivative gives the direction of the conduction velocity vector, and the inverse of the slope in that direction gives the magnitude of the conduction velocity vector. Conduction velocity is anisotropic; it is faster along the direction of tissue fiber orientation and slower perpendicular to it [100]. Therefore, conduction velocity is typically reported as longitudinal (parallel to fiber orientation) and transverse (perpendicular to fiber orientation) conduction velocity. The ratio of longitudinal to transverse conduction velocity is the anisotropic ratio. An increased anisotropic ratio indicates greater ellipticity in the propagating excitation wavefront, and this suggests an increased risk of arrhythmias. From the repolarization phase of the action potential, APD can be calculated. APD is defined as the duration between activation time, defined as above, and repolarization time. Repolarization time is defined as the time at which the cell repolarizes to a set percentage. APD at 20-50% repolarization is used to determine changes in Phase 2 of the action potential, whereas APD at 50-95% is used to determine changes in Phase 3 of the action potential. Beat-to-beat variability in an action potential where every other action potential is similar in morphology or duration is referred to as alternans. Alternans is an arrhythmogenic feature and is typically observed at a faster heart rate and precedes arrhythmias [101, 102].

### Intracellular Calcium

The calcium indicator dyes bind to intracellular calcium in the cytoplasm, and their fluorescence intensity increases when the dyes are in the bound state. Therefore, during a heartbeat, when calcium is released into the cytoplasm from the sarcoplasmic reticulum, the fluorescent signal from the calcium indicators increases, and it returns to baseline during diastole. This change in calcium indicator fluorescence is recorded as a calcium transient. From the calcium transient, the duration of calcium release or rise time, the duration of the entire calcium cycle or calcium transient duration (CaTD), and the time constant of the decay portion or reuptake of calcium can be measured. Calcium alternans or beat-to-beat variability in calcium transient amplitude, morphology, or duration are also observed at faster heart rates [103]. Additionally, when both transmembrane potential and intracellular calcium are measured simultaneously, the delay between the activation of action potential and calcium transient can be measured. This parameter, voltage-calcium delay, is an indicator of the excitation-contraction coupling of the heart.

### Mechanics

Electromechanical optical mapping is a technique where the mechanical deformation of the heart is tracked in addition to electrophysiology [104, 105]. Cardiac mechanics is tracked by tracking the surface geometry of the heart using existing features in the tissue itself and determining the displacement of these geometries over a heartbeat. Fiducial markers can also be applied to the tissue surface to improve motion tracking accuracy, particularly when natural surface features are insufficient or inconsistent [88]. The mechanical deformation is then typically used to reduce the motion-induced artifacts in the fluorescent dye signals.

### Mitochondrial Membrane Potential

Mitochondrial membrane potential is recorded by staining the mitochondrial membrane with tetramethylrhodamine methylester (TMRM) [104]. TMRM is taken up by healthy mitochondria whereas depolarized or apoptotic mitochondria release it. Therefore, TMRM fluorescent intensity correlates with tissue viability, where higher TMRM intensity corresponds to healthy mitochondria and viable tissue and vice versa.

### NADH/FAD

NADH and FAD are autofluorescent molecules which means that when light at a certain wavelength is applied, these molecules emit light at specific wavelengths [21–23]. Therefore, the intensity of the autofluorescent signal corresponds to the total NADH or FAD concentration. However, it needs to be noted that the autofluorescence intensity is also dependent on the amount and intensity of the excitation light that is applied to the tissue. It is, therefore, crucial to apply the same excitation light intensity between experimental conditions where NADH and FAD autofluorescence are compared. The ratio of NADH to FAD is reported as the redox ratio of the tissue [21].

## Maintenance and Troubleshooting

### System Maintenance

The perfusion system should be cleaned routinely between experiments to avoid contamination and salt accumulation. The use of 70% ethanol or 0.1 N hydrochloric acid is recommended for standard procedures to clean the tubing, ECG electrodes, cannula, and stimulation electrode, followed by circulation of deionized water for 30 mins. In instances where contagious agents are used, more aggressive methods involving sterilized and detoxified Etoxaclena detergent (Sigma Chemical Company) and sterile acidic or basic washes have been reported [106]. Tubing must also be regularly replaced in case of wear and tear.

### Common Troubleshooting

#### Terminating a Sustained Arrhythmia

During optical mapping experiments, non-self-terminating arrhythmias may arise. Nonterminating arrhythmias can be extinguished by defibrillation or by injection of 50 mM potassium chloride solution (approximately 500 µl) through the dye injection port [107, 108].

#### Maintaining Appropriate Bath Perfusate Level

In perfusion systems that use peristaltic pumps to circulate perfusates through the Langendorff perfusion system, there can be difficulties in maintaining the perfusate level constant in the tissue bath. Increased bath perfusate levels can decrease optical signal quality by photon scattering. Decreased bath perfusate level risks poor temperature control. A possible cause for difficulty in bath perfusate level is damaged tubing that has increased resistance or decreased pliability (necessary for peristaltic pumps).

#### Inability to Maintain Physiological Bath Temperature

The temperature of the perfusate in the tissue bath is maintained by inserting a heating coil in the bath or by circulating the bath perfusate through a heat exchanger. It is important to record bath temperature over the course of the study to confirm physiological temperature. Changes in room temperature, flow rate, and malfunctioning equipment will need to be addressed to avoid temperature fluctuations in the tissue bath.

#### Motion Artifacts Even in the Presence of Electromechanical Uncouplers

Applying gentle pressure or stretch to physically restrain the tissue without causing ischemia or mechanical damage can help prevent motion artifacts that are observed even after electromechanical uncoupler injection. Additionally, temporarily stopping the circulation of bath perfusate during optical imaging can also help by preventing the disturbance caused by the perfusate flow.

#### Low Signal-to-Noise Ratio

Strategies to improve SNR include increasing dye incubation duration, selecting dyes with faster or more efficient membrane loading, and optimizing dye concentration to avoid toxicity while maximizing signal strength. Stable, high-intensity excitation light sources, such as high-power LEDs, can also enhance signal quality. Improving the efficiency of the optical detection system (i.e., selecting appropriate long-pass or band-pass filters and using sensitive cameras such as CMOS) can significantly increase signal capture. Additionally, minimizing ambient light and optimizing tissue positioning relative to the optical window helps reduce background noise and motion artifacts.

## Limitations and Future Directions Optical Mapping

As discussed in previous sections, the use of fluorescent dyes is critical in optical mapping to record various electrophysiological parameters. However, these dyes, including Di-4-ANEPPS, are often cytotoxic and can cause photodynamic damage and alter electrophysiology [107, 108]. Novel dyes with minimal cytotoxicity are in great demand to reduce damage to cardiomyocytes and changes to electrophysiological activities.

Optical mapping experiments are typically a few hours long due to the deterioration of tissues over time in *ex vivo* conditions. One factor that could contribute to fast tissue degradation is an insufficient nutrient supply (usually glucose) and low oxygen levels in the perfusates. Perfluorocarbon emulsions have been suggested to increase the oxygen-carrying capacity of the perfusates [109]. Such advances could allow longer duration tissue viability and experimental probing in optical mapping experiments.
